# The Absence of Gastrointestinal Redox Dyshomeostasis in the Brain-First Rat Model of Parkinson’s Disease Induced by Bilateral Intrastriatal 6-Hydroxydopamine

**DOI:** 10.1007/s12035-023-03906-7

**Published:** 2024-01-10

**Authors:** Jan Homolak, Mihovil Joja, Gracia Grabaric, Emiliano Schiatti, Davor Virag, Ana Babic Perhoc, Ana Knezovic, Jelena Osmanovic Barilar, Melita Salkovic-Petrisic

**Affiliations:** 1 Department of Pharmacology & Croatian Institute for Brain Research, University of Zagreb School of Medicine, Salata 11, 10 000 Zagreb, Croatia; 2 Interfaculty Institute of Microbiology and Infection Medicine & Cluster of Excellence “Controlling Microbes to Fight Infections,”, University of Tübingen, Tübingen, Germany; 3 Department of Infection and Immunity, Luxembourg Institute of Health, Esch-sur-Alzette, Luxembourg; 4 Faculty of Science, Technology and Medicine, University of Luxembourg, Esch-sur-Alzette, Luxembourg; 5 Faculty of Medicine and Surgery, Department of Biomedical, Metabolic and Neural Sciences, University of Modena and Reggio Emilia, Modena, Italy

**Keywords:** Parkinson’s disease, Gastrointestinal, Gut-brain axis, Oxidative stress, Redox homeostasis

## Abstract

The gut-brain axis plays an important role in Parkinson’s disease (PD) by acting as a route for vagal propagation of aggregated α-synuclein in the gut-first endophenotype and as a mediator of gastrointestinal dyshomeostasis via the nigro-vagal pathway in the brain-first endophenotype of the disease. One important mechanism by which the gut-brain axis may promote PD is by regulating gastrointestinal redox homeostasis as overwhelming evidence suggests that oxidative stress plays a key role in the etiopathogenesis and progression of PD and the gastrointestinal tract maintains redox homeostasis of the organism by acting as a critical barrier to environmental and microbiological electrophilic challenges. The present aim was to utilize the bilateral intrastriatal 6-hydroxydopamine (6-OHDA) brain-first PD model to study the effects of isolated central pathology on redox homeostasis of the gastrointestinal tract. Three-month-old male Wistar rats were either not treated (intact controls; CTR) or treated bilaterally intrastriatally with vehicle (CIS) or 6-OHDA (6-OHDA). Motor deficits were assessed with the rotarod performance test, and the duodenum, ileum, and colon were dissected for biochemical analyses 12 weeks after the treatment. Lipid peroxidation, total antioxidant capacity, low-molecular-weight thiols, and protein sulfhydryls, the activity of total and Mn/Fe superoxide dismutases, and total and azide-insensitive catalase/peroxidase were measured. Both univariate and multivariate models analyzing redox biomarkers indicate that significant disturbances in gastrointestinal redox balance are not present. The findings demonstrate that motor impairment observed in the brain-first 6-OHDA model of PD can occur without concurrent redox imbalances in the gastrointestinal system.

**Supplementary Information:**

The online version contains supplementary material available at 10.1007/s12035-023-03906-7.

## Introduction

Parkinson’s disease (PD) is a chronic progressive neurodegenerative condition characterized by the degeneration of dopaminergic neurons in substantia nigra (SN) pars compacta that results in the development of bradykinesia, tremor at rest, rigidity, and postural instability [1]. Although the etiopathogenesis of the disease remains to be elucidated accumulating evidence points to the involvement of the gastrointestinal tract as (i) the prodromal non-motor symptoms affecting the gastrointestinal tract (e.g., dysphagia, delayed gastric emptying, and constipation) are prevalent and precede the motor phase of PD (sometimes by decades) [2–4]; (ii) a stereotypical spreading pattern of α-synuclein pathology [5, 6] supports the hypothesis that misfolded α-synuclein may originate from the gut [7]; and (iii) mechanistic animal studies clearly demonstrate that pathophysiological events in the gastrointestinal tract are sufficient to trigger and promote the development of the central nervous system (CNS) pathology resembling PD (e.g., [8–11]). Based on the aforementioned evidence a body-first hypothesis of PD has been proposed with the gastrointestinal tract considered the most likely site of early molecular pathophysiological events [12].

In contrast, some studies suggest that in a considerable proportion of patients, PD does not propagate in concordance with the Braak staging system [13, 14]. Furthermore, although highly prevalent, gastrointestinal symptoms are not present in all patients diagnosed with PD, and they do not always appear before the onset of motor symptoms [2, 15]. Consequently, it is evident that in some patients a brain-first hypothesis provides a more accurate explanation of the PD progression.

Based on the aforementioned data, a working model has been proposed which recognizes PD as a complex disease composed of at least two clusters of phenotypes (brain-first and gut-first) [12, 16]. The gut-brain axis plays an important role in both subtypes acting as a route for vagal propagation of aggregated α-synuclein in the gut-first phenotype and as a mediator of gastrointestinal dyshomeostasis via the nigro-vagal pathway in the brain-first phenotype (e.g., [17]). Nevertheless, the mechanisms by which the gut-brain axis may contribute to the propagation of the disease and the appearance of gastrointestinal symptoms remain poorly understood and challenging to study due to overlapping brain and gut pathology in animal models.

In this context, the CNS-targeted 6-hydroxydopamine (6-OHDA) rodent models provide a unique way to study the effects of the brain-first predominant subtype of the disease on the pathophysiological alterations in the gut as the toxin cannot cross the blood-brain barrier. The central 6-OHDA administration model was first introduced by Ungerstedt following the idea that high selectivity of the toxin towards the dopamine uptake sites may result in a specific nigrostriatal dopaminergic lesion [18]. Since its introduction, the model was widely used for investigating many aspects of PD as it successfully recapitulates several important features observed in patients suffering from the idiopathic form of the disease: (i) administration of 6-OHDA mimics increased oxidative stress in dopaminergic neurons found in PD [19, 20]; (ii) SN pars compacta that shows the greatest susceptibility to 6-OHDA-induced injury is also the most affected area in PD patients [21]; (iii) 6-OHDA toxicity can be facilitated with iron [22] and iron dyshomeostasis plays an important role in the etiopathogenesis and progression of PD [23–28]; (iv) 6-OHDA is produced in physiological conditions upon oxidation of dopamine and it is present in the urine of patients suffering from PD. Consequently, it is possible that endogenous 6-OHDA may be involved in the etiopathogenesis of PD in humans [29, 30].

Some groups already utilized the model to study the mechanisms of gastrointestinal dyshomeostasis in the context of the brain-first PD-like nigrostriatal lesion primarily with a focus on gastrointestinal motility (e.g., [31–33]). Other pathophysiological alterations such as decreased expression of the occludin barrier proteins [34] and impaired production of mucus [35] have also been reported.

Interestingly, the effects of brain-first PD-like lesion have, to the best of our knowledge, never been explored in the context of intestinal redox homeostasis regardless of the fact that (i) there is overwhelming evidence that redox dyshomeostasis and oxidative stress play a critical role in the pathophysiology of neurodegeneration [36–38] and the etiopathogenesis of PD [19, 23, 39–42]; (ii) the structure and function of the gastrointestinal barrier and the regulation of redox homeostasis are highly interdependent [43–45]; and (iii) the gastrointestinal tract is considered to be a “free radical time bomb” due to its constant and inevitable exposure to foreign substances and microorganisms with substantial electrophilic potential [45, 46]. Considering that other neurotoxin-based “brain-first” models of neurodegeneration (e.g., the streptozotocin-induced rat model of sporadic Alzheimer’s disease [47] with some resemblance to the 6-OHDA models of PD [48]) demonstrate pronounced redox dyshomeostasis that may contribute to the progression of the disease phenotype [49, 50], we hypothesized that intrastriatal administration of 6-OHDA may produce similar alterations and promote systemic and neuro-inflammation in the rat model of PD contributing to the progression of neuropathology.

The present aim was to measure redox biomarkers along the gastrointestinal tract in a rat model of brain-first PD induced by bilateral intrastriatal administration of 6-OHDA (primary aim) and quantify the effects associated with intrastriatal administration-induced trauma (secondary aim). In most experiments, sham treatment is used as a control procedure; however, the effects of sham treatments are rarely quantified. Here, we included two control groups (intact animals and animals treated intrastriatally with a vehicle) to discriminate between redox responses to 6-OHDA and intrastriatal administration.

## Materials and Methods

### Animals

The experiment was performed on 3-month-old male Wistar rats (*N*=37) from the Department of Pharmacology (University of Zagreb School of Medicine, Zagreb, Croatia). All procedures were approved by the University of Zagreb School of Medicine Ethics Committee and the Croatian Ministry of Agriculture (EP 186 /2018; 380-59-10106-18-111/173) and complied with current institutional, national, and international guidelines (The Animal Protection Act, NN102/17; NN 47/2011; Directive 2010/63/EU).

The rats were housed 2–3/cage with a 7 AM/7 PM 12-h light cycle with controlled humidity (40–70%) and temperature (21–23 °C). Tap water and standardized food pellets were available *ad libitum*, and bedding was changed twice per week. Before the model induction, the animals were randomized into three groups—(i) intact controls (CTR; *N*=9) that would not undergo any procedure (control for the effects of sham procedure); (ii) control animals (CIS; *N*=14) that would receive a bilateral intrastriatal injection of vehicle (control for the effects of 6-OHDA); and (iii) Parkinson’s disease model (6-OHDA; *N*=14) that would receive a bilateral intrastriatal injection of the 6-OHDA solution. The experimental design was unbalanced with respect to the number of animals assigned to each group due to a greater anticipated death rate in the experimental arms with invasive surgical procedures (i.e., CIS and 6-OHDA).

### Intrastriatal Administration of 6-Hydroxydopamine

6-OHDA was dissolved in 0.02% w/v ascorbic acid in sterile saline on the day of the procedure. The animals from the CIS and the 6-OHDA groups were anesthetized by intraperitoneal administration of ketamine (70 mg/kg) and xylazine (7mg/kg). The skin was surgically opened, and the skull was trepanated with a drill. Two microliters of the 6-OHDA solution (4 μg 6-OHDA/μl; 6-OHDA) or an equal volume of vehicle (CIS) was administered into each hemisphere at stereotaxic coordinates: 0 mm posterior, 2.8 mm lateral, and 7 mm ventral relative to bregma and pia mater respectively [51]. The rats were monitored for 24 h after the surgery. The analysis of tyrosine hydroxylase expression was conducted to confirm the successful induction of the model [52].

### Rotarod Performance Test

The rotarod performance test was used to assess motor deficits 3 months after the model induction [53]. Briefly, the animals were first habituated to the task by being placed on the rotating cylinder for 3 days before the test. During the test trial, the animals were placed on an elevated cylinder (diameter = 8 cm) rotating at a constant speed (13 rotations per minute) and the time-to-fall was recorded. Each time-point consisted of equally spaced two consecutive trials with a 180 s cutoff time to account for the possibility that the animal failed to reach the cutoff time due to reasons other than motor deficits. The outcome of the rotarod performance test was defined as cumulative time spent on the rotating cylinder in two 180 s trials.

### Sample Preparation

After 3 months of model induction, the animals were euthanized in deep anesthesia achieved by intraperitoneal administration of ketamine (70 mg/kg) and xylazine (7 mg/kg). Duodenum, ileum, and colon were excised, and luminal contents were removed with a syringe filled with ice-cold phosphate-buffered saline (PBS). The samples were cut open, rinsed again in ice-cold PBS, snap-frozen in liquid nitrogen, and stored at −80 °C until further analyses. Duodenum was sampled 1 cm distal from the pylorus, ileum 1 cm proximal from the cecum, and colon at the midline between the cecum and the sigmoid. The samples were homogenized using an ultrasonic homogenizer (Microson Ultrasonic Cell 167 Disruptor XL, Misonix, Farmingdale, NY, SAD) in 7.5 pH lysis buffer containing 150 mM NaCl, 50 mM Tris-HCl pH 7.4, 1 mM EDTA, 1% Triton X-100, 1% sodium deoxycholate, 0.1% SDS, 1 mM PMSF, protease inhibitor cocktail (Sigma-Aldrich, Burlington, MA, USA), and phosphatase inhibitor (PhosSTOP, Roche, Basel, Switzerland). Homogenates were spun down for 10 min at 4 °C and a relative centrifugal force of 12,879 g, and supernatants were stored at −80 °C. Protein concentration (used as a covariate for other variables to account for differences in lysis efficacy) was measured using the Bradford reagent (Sigma-Aldrich, USA) and bovine serum albumin dissolved in the same lysis buffer for the generation of the calibration curve [54]. The average tissue homogenate protein concentration was 13.7 μg/μl.

### Thiobarbituric Acid Reactive Substances Assay

Thiobarbituric acid reactive substances (TBARS) assay was used to quantify the end products of lipid peroxidation as described in [55]. Twelve microliters of each sample was mixed with 120 μl of the TBA-TCA reagent (w/v 0.4% thiobarbituric acid (Kemika, Croatia) in 15% trichloroacetic acid (Sigma-Aldrich, USA). The samples were diluted with 70 μl of ddH_2_O, vortexed, and incubated in a heating block set at 95 °C for 20 min in perforated microcentrifuge tubes. The reaction was monitored by visual inspection of the experimental and standard samples, and the incubation time was prolonged if needed. The colored product was extracted in 220 μl of n-butanol. The absorbance of the butanol extract was measured at 540 nm in a 384-well plate using the Infinite F200 PRO multimodal plate reader (Tecan, Switzerland). The concentration of TBARS was estimated from the standard curve of the MDA tetrabutylammonium (Sigma-Aldrich, USA) processed in parallel (1–100 μM; *R*^2^=0.998). The extraction procedure was adapted if necessary by adjusting the sample input volume, increasing the duration of the heating step, and modifying the volume of n-butanol used in the extraction procedure based on the partitioning coefficient of the colored product. All adjustments to the procedure were made with standard samples processed in parallel to enable the comparison of standardized estimates.

### Ellman’s Procedure for Determination of Low-Molecular-Weight Thiols and Protein Sulfhydryls

Low-molecular-weight thiols (with glutathione (GSH) being the most abundant) and free protein sulfhydryls (SH) were quantified by measuring 5-thio-2-nitrobenzoic acid (TNB) following the reaction of 5,5′-dithio-bis(2-nitrobenzoic acid) (DTNB) with the low molecular weight and the protein fraction after precipitation with sulfosalicylic acid [49, 56, 57]. Briefly, 25 μl of each homogenate was incubated with 4% w/v sulfosalicylic acid in ddH_2_O for 1 h on ice in a 1:1 volumetric ratio. The samples were centrifuged for 10 min at 10,000 g, the supernatant was dissociated from the pellet, and both were separately reacted with w/v 4 mg/ml DTNB in w/v 5% sodium citrate in ddH_2_O for 10 min. The absorbance of the supernatant from both reactions was read at 405 nm using the Infinite F200 PRO multimodal microplate reader (Tecan, Switzerland), and the concentration was calculated using a molar extinction coefficient of 14,150 M^−1^cm^−1^.

### Superoxide Dismutase Activity

The activity of superoxide dismutase (SOD) was determined indirectly by the inhibition of the 1,2,3-trihydroxybenzene (THB) autoxidation [58, 59] using a modified protocol [57, 60]. The homogenates (3 μl for duodenum or 6 μl for ileum and colon) were placed in a 96-well plate and incubated with a 100 μl of the buffer for measuring total SOD activity (80 μl of the THB solution (60 mM THB dissolved in 1 mM HCl) added to 4000 μl of 0.05 M Tris-HCl and 1 mM Na2EDTA (pH 8.2)) or Fe/Mn-SOD activity ((80 μl of the THB solution (60 mM THB dissolved in 1 mM HCl) added to 4000 μl of 0.05 M Tris-HCl, 1 mM Na2EDTA, 2 mM KCN (pH 8.2)). Absorbance increment at 450 nm reflecting THB autoxidation was measured using the Infinite F200 PRO multimodal microplate reader (Tecan, Switzerland) with 30 s kinetic interval time cycles for 300 s. The purified SOD standard was not used in parallel with the samples so we did not calculate sample SOD unit equivalents to avoid the risk of biased estimates (as the primary aim was to compare groups and not determine the absolute activity of the samples).

### Hydrogen Peroxide Dissociation Rate

Sample-induced H_2_O_2_ dissociation was measured to assess the catalase and residual peroxidase activity using the method originally proposed by Hadwan [61] and modified in [62]. Duodenum (4 μl), ileum (7 μl), and colon (10 μl) samples were first incubated with 100 μl of the Co(NO_3_)_2_ working solution (5 ml of Co(NO_3_)_2_ × 6 H_2_O (0.2 g dissolved in 10 ml ddH_2_O) mixed with 5 ml of (NaPO_3_)_6_ (0.1 g dissolved in 10 ml ddH_2_O) added to 90 ml of NaHCO_3_ (9 g dissolved in 100 ml ddH_2_O)) followed by the H_2_O_2_ working solution (40 μl of 10 mM H_2_O_2_ in 1xPBS) to obtain baseline values for the adjustment of endogenous H_2_O_2_ and/or chemical interference. The same procedure was repeated with samples first incubated with the H_2_O_2_ working solution and the Co(NO_3_)_2_ working solution added at *t*_1_= 60 s to assess the dissociation rate. The H_2_O_2_ concentration in each well was determined from the oxidation rate of cobalt (II) to cobalt (III) in the presence of bicarbonate ions using the carbonato-cobaltate (III) complex ([Co(CO_3_)_3_]Co) absorbance peak at 450 nm with the Infinite F200 PRO multimodal microplate reader (Tecan, Switzerland). The same procedure was repeated with a modified H_2_O_2_ working solution containing 0.025 mM sodium azide (AZD) to inhibit catalase activity (and measure residual peroxidase dissociation rate) [63]. The concentration of H_2_O_2_ was estimated from the standard model obtained by reacting the Co(NO_3_)_2_ working solution with serial dilutions of H_2_O_2_ in 1xPBS. The reaction time and optimal volume of the sample and working solutions were determined based on pilot experiments to ensure the optimal sensitivity of the assay.

### Nitrocellulose Redox Permanganometry

Nitrocellulose redox permanganometry (NRP) [64] was used to determine the total reductive/antioxidative capacity of intestinal samples. Briefly, 1 μl of the homogenate was pipetted onto the nitrocellulose membrane (Amersham Protran 0.45; GE Healthcare Life Sciences, Chicago, IL, USA) and left to dry out at room temperature. The membrane was immersed into the KMnO_4_ solution (0.2 g KMnO_4_ in 20 ml ddH_2_O) for 30 s, rinsed under running dH_2_O, and left to dry out at room temperature. The MnO_2_ precipitate trapped on the membrane was quantified in Fiji (NIH, Bethesda, MD, USA).

### Data Analysis

Data were analyzed in R (4.1.3) in concordance with the guidelines for reporting the evidence from animal studies [65]. The experimenters were not blinded, and the animals were assigned to experimental groups based on stratified randomization (in regard to body mass, home cage allocation, and litter). Survival analysis was done using the *survfit* algorithm from the survival package [66], and the results were reported using *survminer* [67]. The animals were monitored daily since the model induction and all animals that reached the final time-point (85 days) were censored. The group was defined as a stratum. A similar approach was used for the analysis of the rotarod performance test to account for the pronounced ceiling effect as most animals from the control groups (CTR, CIS) reached the cutoff time of 180 s. The cutoff time was not modified during the experiment as the aim was to confirm that the pronounced motor deficit was present in the animals treated with 6-OHDA and the underestimated estimates already informed of a substantial biological effect. The performance was measured as the time spent on the rotating cylinder in two subsequent 180 s trials. Failure to reach the cumulative cutoff time was defined as an *event*, and all animals that reached the cutoff time were censored. The *α* was set at 5%. Redox biomarkers were analyzed using linear regression with the variable of interest corrected for protein concentration used as the dependent variable and group/treatment allocation used as the independent variable. Protein concentration was introduced as an additional covariate in each model to adjust for potential differences introduced during the lysis procedure (i.e., loading control). For THB autoxidation, the difference between the final and baseline absorbance (δTHB) was used as the dependent variable, and baseline absorbance was introduced as a covariate to account for potential chemical interference. For the H_2_O_2_ dissociation rate, the difference in the absorbance between the baseline and the final time-point was used as the dependent variable, and baseline sample absorbance (incubation with the Co(NO_3_)_2_ working solution before the addition of the H_2_O_2_ working solution) was defined as a covariate. Model assumptions were checked using visual inspection of residual and fitted value plots, and transformations were used where appropriate. Model outputs were reported as point estimates with 95% confidence intervals, and differences between groups were reported as ratios of least square means with accompanying 95% confidence intervals. Confidence intervals and *p* values were adjusted using Tukey’s method for multiple comparisons, ensuring the robustness and reliability of the statistical inferences. Univariate models were employed to generate a dataset comprising model-derived estimates of gastrointestinal redox biomarkers, suitable for multivariate exploration to assess latent effects. The dataset was appripriately transformed by centering and scaling and analyzed using dimensionality reduction techniques based on principal component analysis (PCA) and uniform manifold approximation and projection (UMAP).

## Results

### Bilateral Intrastriatal Administration of 6-Hydroxydopamine Induces Pronounced Motor Deficits Confirmed by the Rotarod Performance Test

**Fig. 1:**
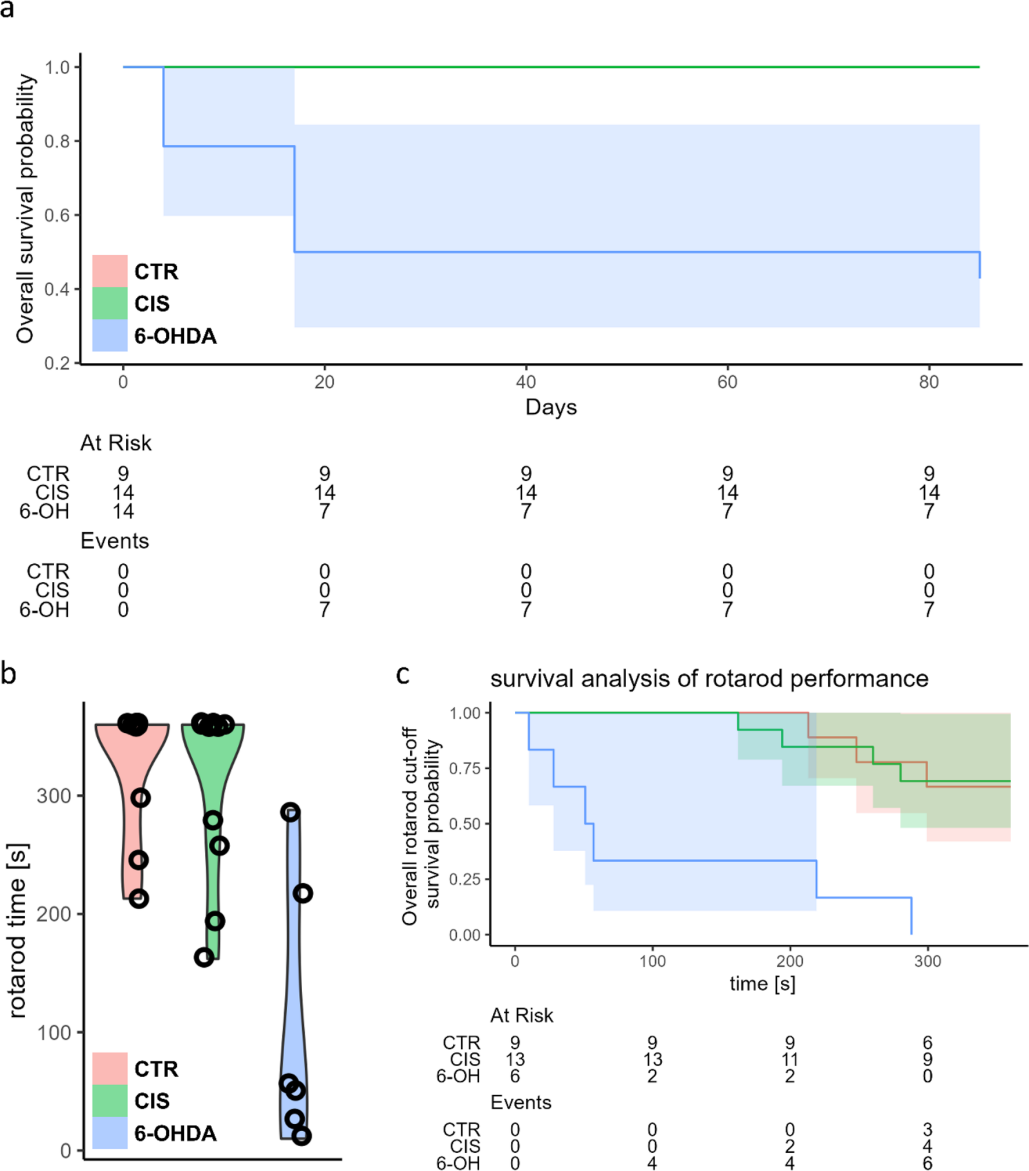
Survival and motor performance of 6-OHDA-treated and the control rats. **A** Survival curve and the risk table for the experiment demonstrate a pronounced dropout rate in the 6-OHDA group. **B** Rotarod performance test 3 months after the model induction demonstrating substantial motor deficits in the 6-OHDA-treated rats. **C** Survival analysis of the rotarod performance test to account for the pronounced ceiling effects in the control groups (CTR, CIS). Time represents cumulative rotarod performance time 3 months after the model induction. CTR intact controls, CIS control animals intrastriatally treated with the vehicle, 6-OH rat model of Parkinson’s disease intrastriatally treated with 6-hydroxydopamine

As expected the 6-OHDA model was characterized by a relatively large dropout rate throughout the experiment while no fatal outcomes were observed in either of the two control groups (CTR, CIS). The fatality rate associated with the 6-OHDA administration was most pronounced in the first month of the experiment (50%) and stabilized thereafter reaching 57% in the final time-point (Fig. 1A). The rotarod performance test showed pronounced motor deficits in the 6-OHDA group and no difference between the intact and the vehicle-treated animals 3 months after the model induction (Fig. 1B, C). Successful model induction was confirmed by western blot and immunohistochemical analysis of tyrosine hydroxylase (TH) expression in the hippocampus, striatum, hypothalamus, and SN (for detailed results please see Knezovic et al. [52]). The expression of TH was significantly reduced in all regions (6-OHDA vs. CIS: −59.8% hippocampus; −52.7% hypothalamus; −82.1 striatum) except in SN where immunofluorescent staining revealed comparable expression of TH in all groups [52] suggesting that slow, progressive, and partial damage spreading from the striatal terminals did not reach SN in 6-OHDA-treated animals by the end of the experiment.

### Motor Deficits Induced by the Bilateral Intrastriatal 6-Hydroxydopamine Are Not Accompanied by Intestinal Redox Dyshomeostasis

**Fig. 2:**
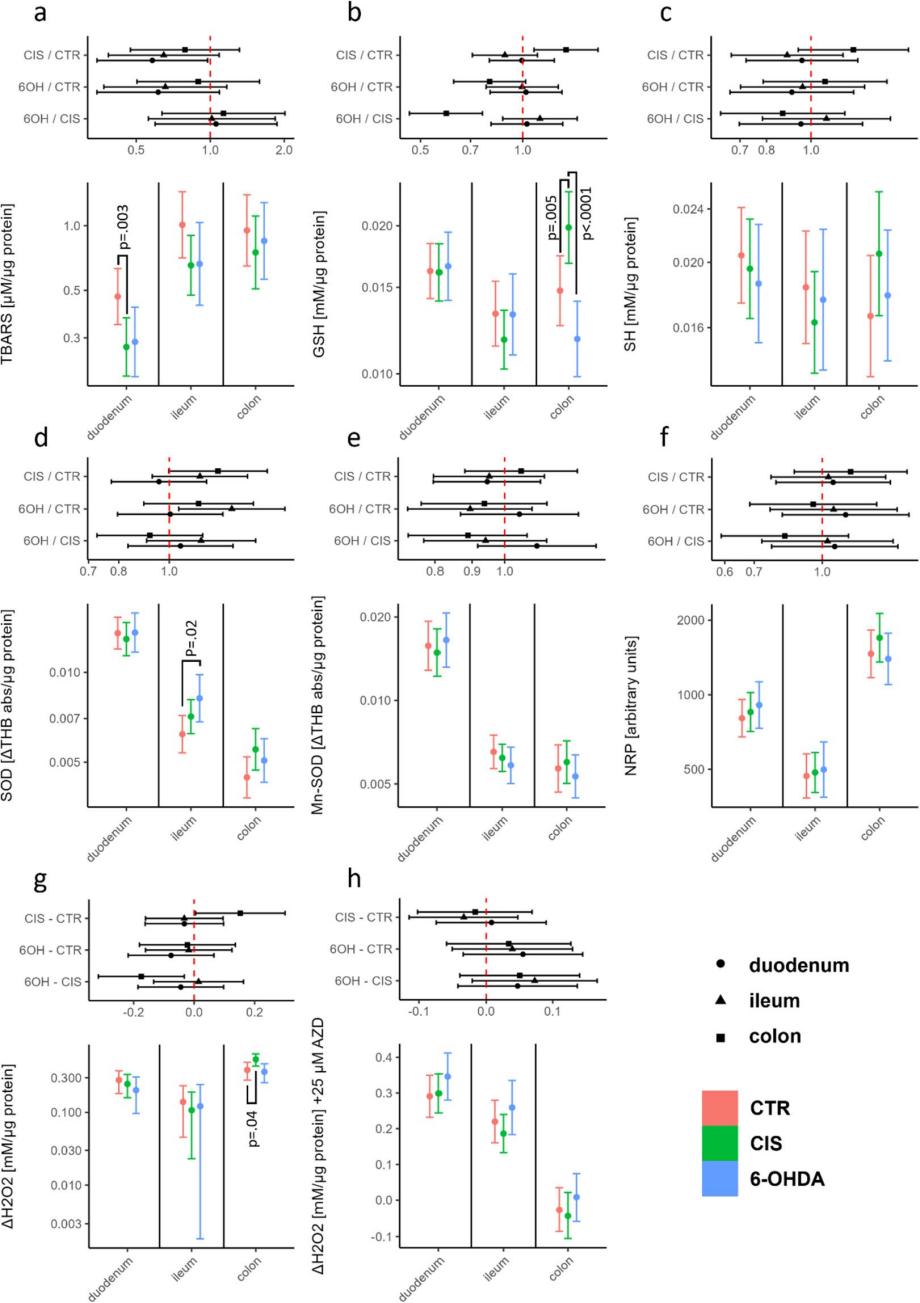
Comparison of redox biomarkers in the control, 6-OHDA, and vehicle-treated animals. **A** TBARS; **B** GSH; **C** SH; **D** SOD; **E** Mn-SOD; **F** NRP; **G** H_2_O_2_ dissociation rate; **H** H_2_O_2_ dissociation rate in the presence of NaN_3_ reflecting the activity of peroxidases. Point estimates of ratios of estimated marginal means with respective 95% confidence intervals are shown. CTR intact controls, CIS vehicle-treated controls, 6-OHDA experimental group treated with bilateral intrastriatal 6-hydroxydopamine, TBARS thiobarbituric acid reactive substances, GSH glutathione, SH free sulfhydryls, SOD superoxide dismutase, AZD sodium azide, NRP nitrocellulose redox permanganometry

Analysis of redox homeostasis in three different segments of the intestine (duodenum, ileum, and colon) indicates that bilateral intrastriatal administration of 6-OHDA is not sufficient to significantly perturb redox homeostasis of the gastrointestinal tract in the rat model of Parkinson’s disease 12 weeks after model induction. Surprisingly, the lipid peroxidation biomarker TBARS exhibited a decrease in the small intestine of animals treated with 6-OHDA (−39% vs CTR (duodenum); −34% vs CTR (ileum)). However, given that a comparable reduction was also observed in sham controls, it is more likely that the observed effect is associated with intrastriatal administration rather than changes resembling motor dysfunction in PD (Fig. 2). In sham controls, there was also an elevation in GSH content (+34% vs CTR) and H_2_O_2_ dissociation capacity (+40% vs CTR) observed in colon tissue, whereas both effects were absent in the 6-OHDA group. The only discernible alteration in a redox biomarker within the gastrointestinal tract of 6-OHDA-treated animals, as compared to controls, was the activation of SOD in the ileum (+32% vs CTR). However, given the absence of similar changes in closely related redox biomarkers and in neighboring anatomical areas, the observed result might be indicative of a type I error.

**Fig. 3:**
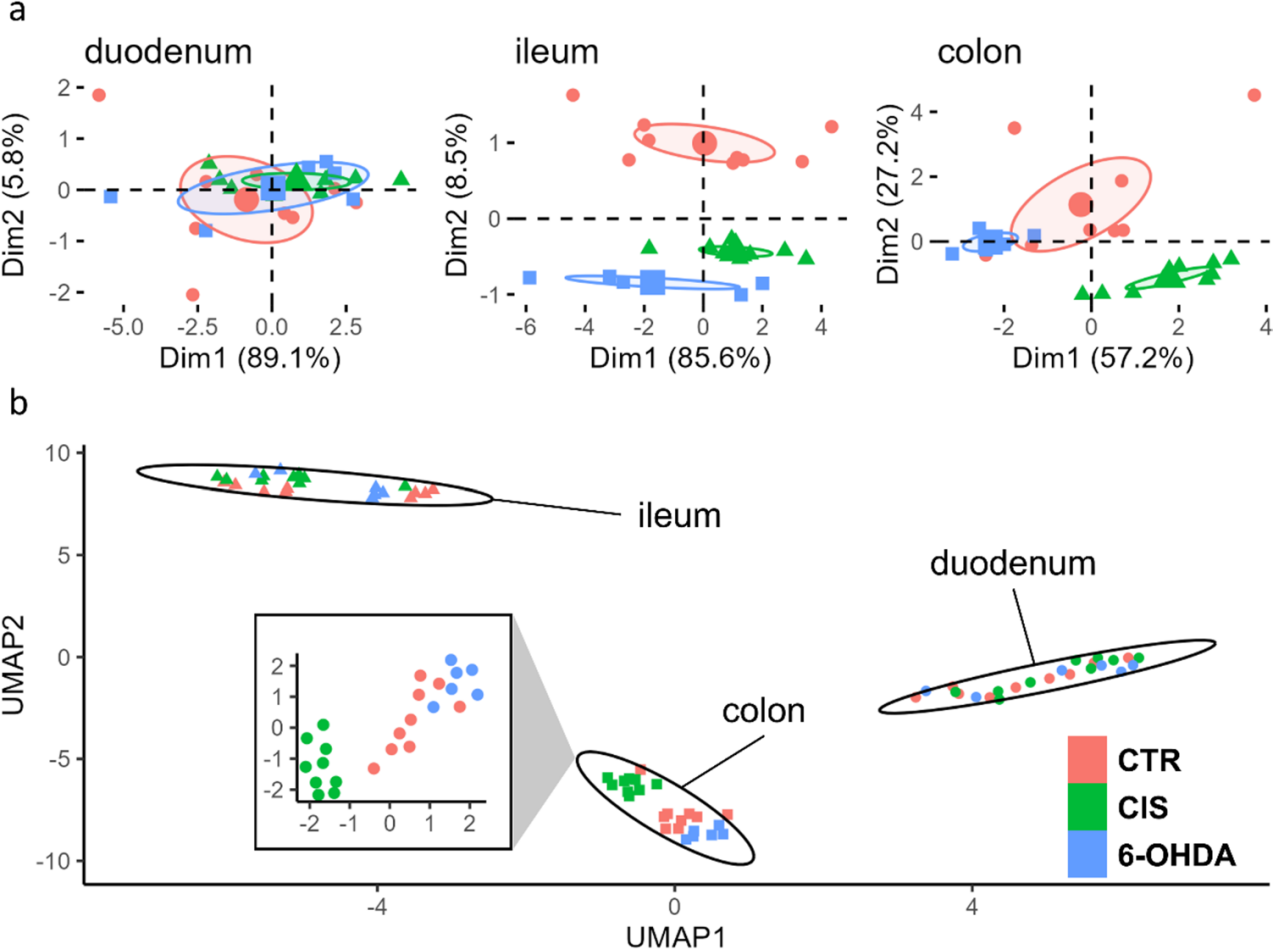
Multivariate exploratory analysis of PCA on redox-related biomarkers in the duodenum, ileum, and colon of the brain-first 6-OHDA-induced rat model of PD. **A** Biplot illustrating the clustering of animals based on the first two principal components in the duodenum, ileum, and colon. **B** Visualization of the uniform manifold approximation and projection space depicting a distinct separation between anatomical segments, with notable clustering of groups within the colon space. Notably, redox biomarkers from the sham-operated animals exhibit the most pronounced level of distinction. PCA principal component analysis, CTR control, CIS intrastriatally treated controls, 6-OHDA 6-hydroxydopamine, PD Parkinson’s disease, Dim1 1^st^ dimension, Dim2 2^nd^ dimension, UMAP uniform manifold approximation and projection

The model-derived estimates were then analyzed using PCA and UMAP for a comprehensive exploration of effects that might have been concealed by subtle influences and the intricate biology of redox regulation. The first PCA-derived dimension captured a substantial amount of variance, accounting for 89%, 86%, and 57% in the duodenum, ileum, and colon, respectively (Fig. 3; Supplementary material). Notably, the second component of redox biomarkers in the ileum (influenced by SOD and TBARS) distinguished between sham controls and 6-OHDA animals, although the impact was modest, as the second dimension only captured 8.5% of the variance. Conversely, PCA of redox biomarkers in the colon facilitated differentiation between 6-OHDA and sham-operated animals, primarily represented by NRP, GSH, and SH (Fig. 3, Supplementary material). UMAP-based clustering provided a clear separation between anatomical segments; however, the distinction between groups was only apparent in the colon, where sham-operated animals exhibited a pronounced separation.

## Discussion

The presented results provide solid evidence against the existence of pronounced gastrointestinal redox dyshomeostasis in a brain-first rat model of PD induced by bilateral intrastriatal administration of 6-OHDA 12 weeks after model induction. Although the possibility that redox dyshomeostasis was present but too subtle to be estimated with a high degree of certainty using the current experimental design and methodological approach cannot be completely excluded, a substantial dropout rate and pronounced motor deficits (Fig. 1) accompanied by unconvincing alterations of eight individual redox biomarkers in three separate anatomical regions (Figs. 2 and 3) strongly speak against the presence of gastrointestinal redox dyshomeostasis and/or its biological significance. The latter is further supported by the fact that the same biomarkers have been shown to be sufficiently sensitive to detect redox-related alterations induced by relatively mild stimuli (e.g., single orogastric administration of the 200 mg/kg D-galactose solution) [57] and in other transgenic [68] and neurotoxin-based models of brain-first-induced neurodegeneration [49, 50]. Multivariate analysis was employed to assess redox homeostasis, considering the potential presence of subtle yet consistently congruent and biologically plausible alterations. These changes might not have been observed in univariate models due to the limited sensitivity of certain methods to minute changes and/or the apparent biological variability. Despite utilizing multivariate analysis techniques such as PCA and UMAP, it was observed that gastrointestinal tissue samples from 6-OHDA animals could not be distinctly differentiated from those of intact and/or sham-operated controls. This lack of differentiation based on the relationships between redox biomarkers, was indicative of the absence of meaningful redox dyshomeostasis.

The data presented here seem to be in contrast with the results reported by Pellegrini et al. who found evidence supporting increased lipid peroxidation in the colon of the 6-OHDA-treated rats 4 and 8 weeks after model induction in the only other study in which oxidative stress-related biomarkers were measured in the gastrointestinal tract of the brain-first 6-OHDA-induced model of PD [69]. Nevertheless, the apparently discrepant results may be explained by several methodological differences. Pellegrini et al. used the 6-OHDA model in which the toxin is injected unilaterally into two sites of the medial forebrain bundle (MFB) in a total dose of 7.5 μg/3 μl [69], and we used bilateral intrastriatal administration of 8 μg/2 μl into each hemisphere to overcome potential compensatory mechanisms of the un-lesioned hemisphere that may mask some of the effects of the nigrostriatal dopaminergic neurodegeneration [70]. Both the dose and the site of injection have a profound effect on the model phenotype and spatiotemporal patterns of dopaminergic neurodegeneration. While administration of 6-OHDA into the SN and/or the MFB induces a complete and rapid lesion of the nigrostriatal pathway in hours, striatal injection usually produces a slow, progressive, and partial damage spreading from the striatal terminals to SN for weeks [71]. Considering that in our experiment SN lesion was minimal even after 12 weeks (assessed by control SN TH immunopositivity [52]), it is possible that the absence of the effects in the gut observed here was due to relative preservation of the nigro-vagal pathway (the main pathway implicated in the gut-related PD symptoms [72]). Furthermore, Pellegrini et al. measured only a single redox biomarker—malondialdehyde (MDA) using the TBARS method [69]. Although TBARS is a widely used and reliable biomarker of oxidative stress [73] its use has many caveats and limitations, and it has been proposed that it can offer “at best, a narrow and somewhat empirical window on the complex process of lipid peroxidation” [74]. MDA is one of many different aldehydes that are produced as secondary products of lipid peroxidation, and its concentration inside cells depends on many biological processes [73, 75]. Importantly, an increased concentration of MDA can sometimes reflect the activation of protective antioxidant defense systems in case its metabolism and the rest of the redox regulatory network work correctly [75]. Considering that MDA is also produced in the process of prostaglandin biosynthesis, especially the synthesis of thromboxanes via the thromboxane synthase [73] implicated in the regulation of inflammation in the gastrointestinal tract [76], it is possible that increased colonic MDA reported by Pellegrini et al. was more related to the observed pro-inflammatory signaling (increased tumor necrosis factor α and interleukin-1β [69]) than a complete failure of the redox homeostasis resulting in uncontrolled peroxidation of membrane lipids. Nevertheless, inflammatory and redox processes demonstrate a high level of biological interdependence [77] so in the context of pro-inflammatory alterations reported by Pellegrini et al. redox dysregulation can by no means be excluded. Finally, Pellegrini et al. measured TBARS in “colonic neuromuscular tissue,” and here we measured redox homeostasis in the intact whole preparations to (i) overcome potential bias introduced by dissection and sample preparation; and (ii) take into account alterations of the mucosa due to its critical importance for the maintenance of the redox homeostasis in the gastrointestinal tract [78]. It also cannot be excluded that gastrointestinal oxidative stress may be present only in the early stages of 6-OHDA-induced pathology (detected by Pellegrini et al. [69]) and wane over time (i.e., until the 3-month time-point presented here).

Considering that oxidative stress plays a key role in PD [23, 79], and that accumulating evidence supports the involvement of the gastrointestinal tract (a “free radical time-bomb” [45, 46]) in the etiopathogenesis and progression of the disease, it is surprising that primary (i.e., in the context of gut-first models) and secondary (subsequent to CNS degeneration in the brain-first models of PD) gastrointestinal dysfunction has never been thoroughly explored in the context of gastrointestinal, systemic, and brain redox dyshomeostasis. The results presented here provide evidence for the absence of secondary contribution of gastrointestinal oxidative stress in the brain-first 6-OHDA model of PD (at least in the period in which there is no sufficient nigro-vagal degeneration to induce peripheral dyshomeostasis); however, future research should elucidate the contribution (or the lack of thereof) of the gastrointestinal tract to oxidative stress in other animal models of PD (especially in the gut-first models) and finally—in patients. The importance of understanding the contribution of gastrointestinal stress to oxidative stress in the context of PD is also evident from a recent *Drosophila* study by Liu et al. who demonstrated that overexpressing α-synuclein exclusively in the gut is sufficient to recapitulate the phenotypic traits of PD and that intestinal generation of free radicals was the main mediator of the observed phenomenon [11].

## Conclusion

In conclusion, by measuring eight specific redox biomarkers in different regions of the gastrointestinal tract (duodenum, ileum, and colon), we found that the bilateral intrastriatal administration of 6-OHDA leads to significant motor impairments (as observed in the rotarod test) but does not affect the redox balance in the gastrointestinal system. However, it is important to interpret these results in light of the fact that substantial damage to SN did not occur in this experiment. The literature recognizes the nigro-vagal pathway as a crucial contributor to gastrointestinal dysfunction in PD. It is possible that disturbances in gastrointestinal redox homeostasis may arise after SN degeneration. Therefore, when studying gastrointestinal symptoms associated with PD using brain-first 6-OHDA models, it is essential to carefully consider the dosage and injection site of 6-OHDA, as they can have significant impacts on the development of pathophysiological changes in the gastrointestinal tract.

## Limitations

Finally, several limitations of the present work have to be emphasized. In the present study, there was a pronounced dropout rate in the 6-OHDA-treated group of animals (57% fatality rate) which may have introduced attrition bias—e.g., the animals with the most pronounced response to the 6-OHDA administration and possibly more rapid spreading of the 6-OHDA-induced damage from the striatal terminals to SN that may have developed gastrointestinal redox dyshomeostasis were excluded from the study as they died before the final time-point. Furthermore, the main aim of the study was to assess redox homeostasis so additional functional gastrointestinal parameters were not measured. Animal models of PD often demonstrate gastrointestinal dysmotility (hypothesized to be mediated by the degeneration of the nigro-vagal pathway [72]) so the association (or the lack of thereof) between gastrointestinal dysmotility and gastrointestinal redox homeostasis remains to be explored in future studies. The latter seems particularly interesting as redox disbalance has been recognized as an important regulator of gastrointestinal motility [80, 81] and it has been hypothesized that motor activity of the gastrointestinal tract may modulate gastrointestinal redox homeostasis [82]. Gastrointestinal redox homeostasis may also be influenced indirectly by behavioral and metabolic consequences of 6-OHDA administration (e.g., altered circadian activity and feeding patterns). Considering that neither circadian activity nor feeding patterns were monitored in the present study and included in statistical models it cannot be excluded that unavoided bias at this level may have masked some of the effects. Future studies should take into account the possible indirect effects of 6-OHDA in this context as well. In this study, 6-OHDA was administered to relatively young rats to mimic the PD model used by most researchers because 6-OHDA induces dopaminergic lesions regardless of age. The results may have been different in old animals as it has been shown that older rats demonstrate a higher susceptibility to 6-OHDA toxic insult [83], α-synuclein propagation, depletion of neurotransmitters, microbiome changes, etc. [84]. Furthermore, most researchers use unilateral 6-OHDA injection to model PD, and here we used bilateral administration to avoid missing the peripheral effects due to compensatory action of the undamaged side. Although there are differences between unilateral and bilateral 6-OHDA models [70], the absence of gastrointestinal redox dyshomeostasis in the bilateral model provides rational evidence to assume the effects would also be absent if the rats were injected unilaterally. Lastly, none of the redox biomarkers we evaluated here supported the hypothesis of gastrointestinal redox dyshomeostasis; however, the area of aging-related redox biomarkers is developing rapidly, and it is possible that the analysis of some other (more sensitive) biomarkers or signaling molecules (e.g., redox-sensitive modulatory proteins) [85–87] may have provided a different insight.

### Supplementary Information

ESM 1 (DOCX 169 kb)

## Data Availability

Raw data can be obtained from the corresponding author. The manuscript has been preprinted on bioRxiv [88].
