# Supplementary material for: The Absence of Gastrointestinal Redox Dyshomeostasis in the Brain-First Rat Model of Parkinson’s Disease Induced by Bilateral Intrastriatal 6-Hydroxydopamine
Source: Mol Neurobiol. 2024 Jan 10;61(8):5481–93. doi: 10.1007/s12035-023-03906-7 (PMC11249596; doi:10.1007/s12035-023-03906-7)
Supplement: Supplementary file 1 — (DOCX 169 kb) [file 12035_2023_3906_MOESM1_ESM.docx]

Supplementary figures for: The absence of gastrointestinal redox dyshomeostasis in the brain-first rat model of Parkinson's disease induced by bilateral intrastriatal 6-hydroxydopamine (Homolak et al. 2023)


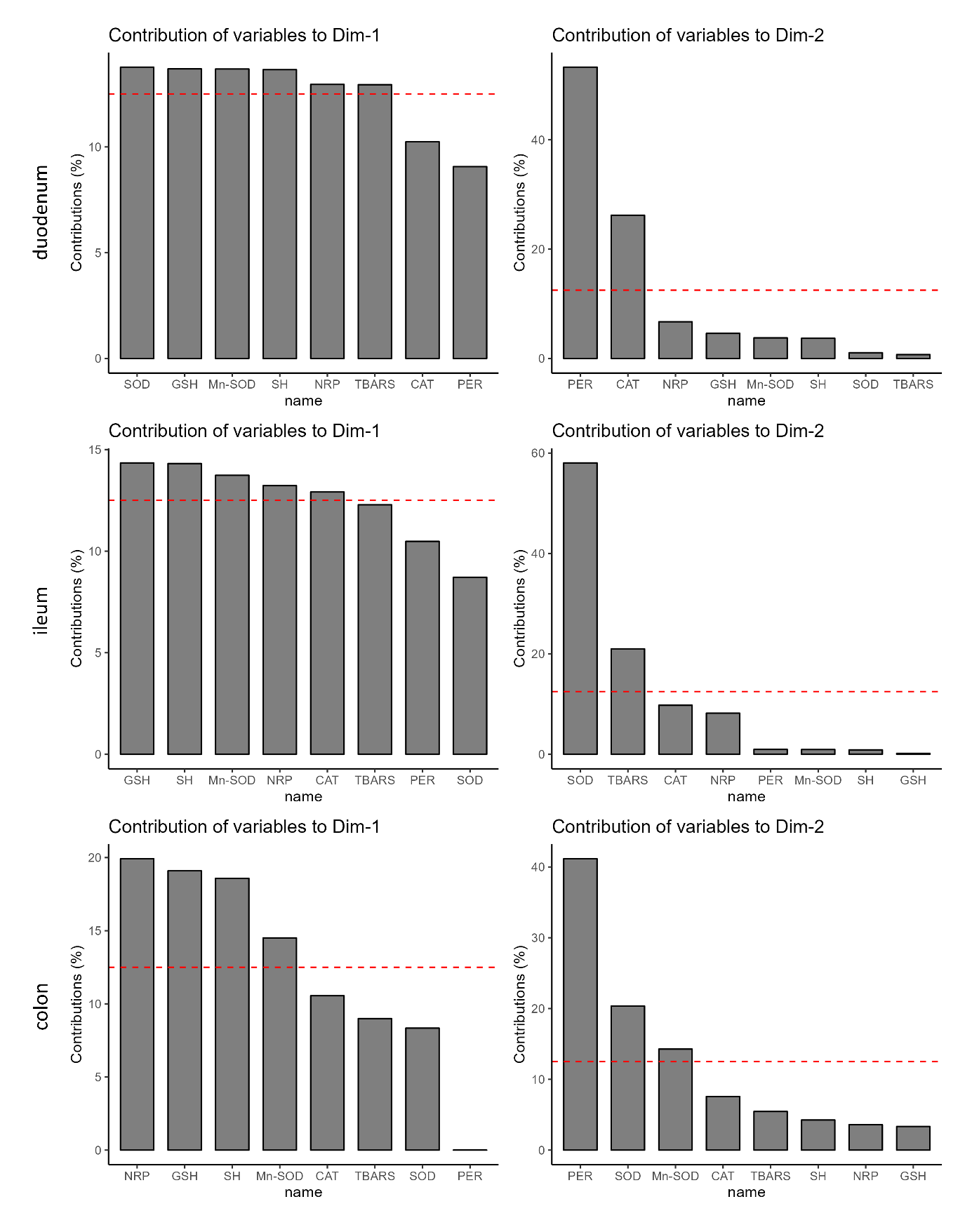


**Fig S1.** Contribution of variables to the first two principal components. PER – peroxidese (NaN_3_ resistant H_2_O_2_ dissociation rate); SOD – superoxide dismutase; CAT – catalase (H_2_O_2_ dissociation rate); TBARS – thiobarbituric acid reactive substances; SH – protein sulfhydryl residues; NRP – nitrocellulose redox permanganometry; GSH – glutathione (low molecular weight thiols).
